# Chronic Hepatitis B Prevalence among Children and Mothers: Results from a Nationwide, Population-Based Survey in Lao People's Democratic Republic

**DOI:** 10.1371/journal.pone.0088829

**Published:** 2014-02-28

**Authors:** Anonh Xeuatvongsa, Kenichi Komada, Tomomi Kitamura, Phengta Vongphrachanh, Chansay Pathammavong, Kongxay Phounphenghak, Thongchanh Sisouk, Darouny Phonekeo, Bounthanom Sengkeopaseuth, Vilasak Som-Oulay, Koji Ishii, Takaji Wakita, Masaya Sugiyama, Masahiko Hachiya

**Affiliations:** 1 National Immunization Program, Ministry of Health, Lao PDR, Simeuang Road, Vientiane, Lao PDR; 2 Bureau of International Cooperation, National Center for Global Health and Medicine, Shinjuku, Tokyo, Japan; 3 National Center for Laboratory and Epidemiology, Ministry of Health, Lao PDR, Simeuang Road, Vientiane, Lao PDR; 4 Department of Virology II, National Institute of Infectious Diseases, Musashi-murayama, Tokyo, Japan; 5 Hepatology Research Center, National Center for Global Health and Medicine, Ichikawa, Chiba, Japan; CEA, France

## Abstract

**Background:**

Hepatitis B is regarded as a serious public health issue in Lao People's Democratic Republic (Lao PDR), a Southeast Asian country. However, disease epidemiology among the general population is not well known, and thus a nationwide cross-sectional survey for hepatitis B surface antigen (HBsAg) prevalence in children and their mothers was conducted.

**Methods and findings:**

We applied three-stage cluster sampling using probability proportionate to size. After randomly selecting child (5 to 9 years old) and mother (15 to 45 years old) pairs from the selected villages, questionnaires and HBsAg rapid tests were conducted. Data from 965 child and mother pairs were analyzed. Multivariate logistic regression analyses were used to investigate the independent association of individual background characteristics for the odds of being HBsAg positive. In total, 17 children and 27 mothers were HBsAg positive. HBsAg prevalence was estimated to be 1.7% (95% confidence interval: 0.8%-2.6%) in children, and 2.9% (95% confidence interval: 1.7%–4.2%) in their mothers after taking sampling design and weight of each sample into account. Mother's infection status was positively associated with HBsAg positivity in children (*p*<0.001), whereas other potential risk factors, such as ethnicity, proximity to health centers, and history of surgery, were not. There were no significant associations between mother's HBsAg status and history of surgery, and other sociodemographic factors.

**Conclusions:**

Despite the slow implementation of the hepatitis B vaccination program, HBsAg prevalence among children and their mothers was not high in Lao PDR compared to reports from neighboring countries. The reasons for the differences in prevalence among these countries are unclear. We recommend that prevalence surveys be conducted in populations born before and after the implementation of a hepatitis B vaccination program to better understand the epidemiology of hepatitis B.

## Introduction

More than two billion people have been infected with hepatitis B worldwide, and among these individuals, more than 350 million suffer from chronic hepatitis B virus (HBV) infection [1], [2], [3]. Infection with HBV results in 600,000 to 1.2 million deaths per year due to chronic hepatitis, cirrhosis, and hepatocellular carcinoma [2], [4]. HBV is responsible for 60% to 80% of the world's hepatocellular carcinoma cases, one of the major three causes of death in Africa, Asia, and the Pacific Rim, and accordingly, has been categorized as a Group 1 carcinogenic agent to humans by the International Agency for Research on Cancer [5].

The prevalence of hepatitis B differs throughout the world. Southeast Asian countries have been estimated to have a chronic HBV infection rate of more than 8% before the introduction of hepatitis B vaccination [6]. The Western Pacific region of the World Health Organization (WHO), to which most of the Southeast Asian countries belong, is assumed to have a high prevalence of hepatitis B [7]. Specifically, the prevalence is estimated to be 9% to 12% among women of childbearing age [8] and 8% to 10% among children in pre-vaccine era [9]. The WHO estimates that the region has 28% of the global population, while it accounts for almost half of all chronic hepatitis B infections worldwide [10].

Hepatitis B vaccination, especially within 24 hours after childbirth, is considered the most effective and efficient preventive measure against hepatitis B infection [3], [11]. Based on these assumptions, the WHO set goals to lower the prevalence of chronic hepatitis B among children over 5 years of age to 2% by 2012 and 1% by 2017. To achieve these goals, the WHO plans to increase immunization coverage to 65% for the birth dose and 80% for the third dose of the hepatitis B vaccine [7].

Lao People's Democratic Republic (Lao PDR) is a Southeast Asian country, located in the center of the Indochina peninsula. The country is landlocked and surrounded by China, Vietnam, Cambodia, Thailand, and Myanmar. The neighboring countries report relatively high hepatitis B prevalence compared to other parts of the world. For example, a survey from two provinces in Cambodia reported a hepatitis B surface antigen (HBsAg) prevalence of 7.7% (95% CI: 6.2%–9.3%) among healthy volunteer adults [12]. Another population-based survey in a province in rural Vietnam found that 18.8% (95% CI: 15.7%–21.9%) of adults and 12.5% (95% CI: 9.7%–15.3%) of infants were HBsAg positive at the time of the survey [13]. Thus, Lao PDR has been regarded as one of the hyperendemic countries for hepatitis B for quite some time and is ranked as a priority country by the WHO [7], [9] despite a lack of data on the prevalence in a representative population. Pre-vaccine era prevalence was estimated as 11.8% [4], 8–10% [9], or 8% or more [6] for Lao PDR and Indochina countries. In response to this situation, Lao PDR has implemented the hepatitis B vaccine into the routine immunization program since 2002 (at 6, 10, and 14 weeks after birth), as well as birth dosing since 2004. The birth dosing was initiated at referral hospitals in the capital city, and then gradually expanded into rural hospitals (2006), and eventually home deliveries (2010). However, since then, no direct investigation has been conducted, and thus a nationwide survey is warranted [7], [9]. The routine immunization coverage is reported as 56% for BCG, 50% for the third DPT, 50% for the third hepatitis B, 40% for measles, and 46% for oral polio vaccine in 2007, when a proportion of target children were born [14].

The primary objective of the present study was to estimate the chronic HBV infection rates by measuring the seroprevalence of HBsAg among children aged 5 to 9 years, and their mothers aged 15 to 45 years.

## Methods

### Ethical considerations

The survey protocol was reviewed and approved by the Ethical Committee of the Ministry of Health, Lao PDR, and the institutional review board of the National Center for Global Health and Medicine, Japan (NCGM-G-001130-00). Access to selected households was granted by the Ministry of Health, and the provincial and district government authorities.

After obtaining approval to conduct the survey from local authorities, surveyors explained the purpose of the survey to village leaders, selected participants, and their caregivers, assured them that all information would be strictly confidential and that no names would be gathered, and that there would be no benefit or penalties for agreeing or refusing to participate. Written informed consent was obtained from each mother on behalf of her child for each pair. Written informed consent was obtained from legal representatives (next of kin, caregivers, or guardians) when mothers were illiterate. The respondents' names were not recorded on the questionnaire sheets.

### Study population

The target population was children aged 5 to 9 years (date of birth: January 2, 2002 to January 1, 2007) and their mothers aged 15 to 45 years (date of birth: January 2, 1966 to January 1, 1997) living in the selected cluster at the time of the survey. The reasons for this selection criteria are: 1) the national and regional hepatitis control policy target is to reduce chronic hepatitis B prevalence among children aged 5 years or older [7]; 2) Lao PDR does not have reliable HBsAg prevalence data among healthy adults, and mothers of childbearing age are considered the major source of hepatitis B infection for children; and 3) our pilot survey revealed that between 20 and 25 mother and child pairs can be practically sampled from each village.

### Calculation of sample size

The equation used to calculate the required sample size is as follows [15], [16]:
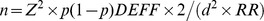
where *n* = sample size

*Z* = significance level for 95% confidence

*p* = expected prevalence

*DEFF* = design effect

*d* = precision

*RR* = response rate

The sample size (*n*) of 961 was calculated on the basis of an expected HBsAg seroprevalence (*p*) of 5%, a 5% level of significance (*Z*), precision (*d*) of ±2.0%, design effect (*DEFF*) of 2.0, two strata, and response rate (*RR*) of 95%. For field practicability, we requested 24 survey teams to sample 21 child and mother pairs from each cluster, with the aim of gathering 1,008 pairs in total.

### Survey design and sampling

The survey applied a stratified three-stage random cluster sampling design, a type of probability sampling recommended by the WHO [15], [17]. The survey was carried out by 24 survey teams (two members per team). Team members were recruited from the same districts that were under investigation to implement the survey more smoothly. The survey teams consisted of epidemiology, surveillance, or laboratory staff. The survey teams were supervised by 11 national personnel (six from the National Immunization Program and five from the National Center for Laboratory and Epidemiology, Ministry of Health) as well as 13 provincial officers.

For stratified multistage cluster sampling, immunization coverage by district and population data were obtained from the National Immunization Program, the Ministry of Health, and the Department of Statistics, Lao PDR. For post-survey weight adjustment, the survey teams obtained the latest population data from village leaders or health volunteers.

All 143 districts in Lao PDR were stratified into two strata, one having high (more than 76%) and the other having low (76% or less) immunization coverage for the third diphtheria, pertussis, tetanus, and hepatitis B (DPT-HepB)vaccines as reported in 2010. For the first stage, we selected 12 districts from each stratum using probability proportionate to size (PPS) sampling based on the population census of 2005. For the second stage, we selected two villages from each selected district by PPS sampling, and 48 villages were randomly sampled in total. In the instances in which the selected village lacked a sufficient number of children or the survey team could not approach the selected village due to safety or security reasons, the nearest village on the way back to the district center was selected. For each selected village, surveyors obtained a list of households, including age and sex, primarily from the poverty reduction program data with the assistance of the village leader, women's union, and/or healthcare volunteer. From these lists, 21 mothers aged 15 to 45 years old with children aged 5 to 9 years were randomly selected using a paper-based lottery system. When a mother had multiple children aged 5 to 9 years old, the youngest child was chosen for the survey. Special attention was paid to ensure that the child's biological mother was surveyed, as adoption is common in rural Lao PDR.

The survey was carried out from January 25^th^ to February 4^th^, 2012. Each survey team successfully approached their assigned villages, with the exception of one village, which could not be visited because of road difficulties. An alternative village was chosen according to the predetermined selection criteria. In total, 1,008 children and 1,008 mothers were sampled. The overall response rate for HBsAg was 100%; however, 43 pairs were excluded from the analysis due to age ineligibility. That is, one child was over 9 years of age and 33 were less than 5 years of age. Furthermore, three mothers were over 45 years of age and six were less than 15 years of age. This happened as 43 mothers confused calendar age with traditional age. In rural areas, newborns start at one year old and a year is added to their age for each passing of a Lunar New Year. The surveyors asked participants for their age in years and their date of birth, and checked that they matched. A total of 965 pairs were included for analysis.

### Questionnaires

A brief face-to-face questionnaire was administered to the sampled mother. The questionnaire consisted of 25 questions in four domains of inquiry: sociodemographic background of the family (i.e., ethnicity, family head's occupation, and mother's education level), family history of liver diseases, including mother, demographic characteristics of the child (i.e., age, sex, and place of birth), and immunization records. Additionally, questions were asked regarding exposure to potential risk factors for acquiring hepatitis B infection (e.g., history of blood transfusion, surgical operation, and sharing of toothbrush). The questionnaire was developed in English, translated into Lao, back-translated into English, and then compared and revised by bilingual staff members. A small pilot test was conducted prior to the data collection.

### Testing for HBsAg

We used a simple and rapid test (Alere Determine HBsAg test card; Alere Medical Co. Ltd., Chiba, Japan) rather than the traditional ELISA test, as it was better suited to use in the field [14]. The sensitivity and specificity of the test were reported as high in two Asian countries [18], [19]. In Vietnam, the Determine HBsAg test validity was measured based on comparison with HBsAg EIA. Results were 100% in both sensitivity and specificity in 328 samples [18]. In China, the Determine HBsAg performance was evaluated in comparison with HBsAg EIA for 671 samples. The sensitivity was reported to be 98.9% and specificity 100% [19]. The Determine HBsAg examination kit is one of the most reliable point-of-care HBsAg tests, and is recommended by the WHO [15]. HBsAg testing was performed according to the manufacturer's instructions. Blood was collected from a finger prick using a safety lancet (BD Safety Lancet, Becton Dickinson, NJ, USA) and glass capillary tube, and the blood was applied onto the sample pad of the rapid test kit. After applying the chase buffer, surveyors assessed the results after at least 15 minutes, but no longer than 24 hours. When no control bar appeared after 15 minutes, the test results were considered invalid, and the test was repeated. Blood spots were collected onto filter paper for further testing. A 2-day training session was organized for surveyors and supervisors on the use of the rapid test and the completion of the questionnaire. To ensure the safety of the blood collection procedure, surveyors always used a new pair of latex gloves. Surveyors were instructed to place all capillary tubes and lancets into safety boxes immediately after use.

### Data entry and statistical analysis

All of the completed questionnaires were brought to a centralized location and the data were entered into a Microsoft Excel 2007 spreadsheet. Data were double-entered and cross-checked. Logistic regression tests and odds ratios were used to examine the relationship between the independent variables and HBsAg results. Multivariate logistic regression was used to investigate the independent association of different household and individual characteristics with the odds of being HBsAg positive. All estimates and standard errors were calculated by taking the multistage clustered sampling design and the weight of each sample into account to give representative, unbiased results. A *p* value <0.05 was considered statistically significant.

In our regression analyses, we adjusted for potential confounders by using the following variables: third DPT-HepB immunization coverage at the location of current residence, mother's age, ethnic group, mother's education level, family head's occupation, and mother's HBsAg status. For multivariate logistic regression analyses, multicollinearity was tested by calculating the variance inflation factors for each independent variable, and a value of more than 10 was considered as having multicollinearity.

All statistical analyses were carried out using STATA version 12 (Stata Corp., College Station, TX). Means and proportions were calculated using STATA's ‘svy’ function, with each sample weighted according to estimated population size.

## Results

### Socioeconomic backgrounds

The baseline characteristics of the 965 mothers and their children are summarized in Table 1. The mean age of the mothers was 29.1 years (95% CI: 26.2–33.1), and the mean age of the children was 5.8 years (95% CI: 5.4–6.3). Of the sampled children, 474 (49.4%) were male and 486 (50.6%) were female (five were unknown).

**Table 1:** HBsAg prevalence among children (5 to 9 years old) and mothers (15 to 45 years old) in Lao PDR by selected background characteristics.

		n	%	Children's HBsAg (+)	%	95% CI	Mothers' HBsAg (+)	%	95% CI
Mothers' age(n = 965)	15–19	4	0.41	0	0.00		0	0.00	
	20–24	85	8.80	1	1.18	0.00–3.52	3	3.53	0.00–7.53
	25–29	294	30.47	7	2.38	0.63–4.13	8	2.72	0.85–4.59
	30–34	275	28.50	6	2.18	0.44–3.92	9	3.27	1.16–5.39
	35–39	176	18.24	3	1.70	0.00–3.64	3	1.70	0.00–3.64
	40–45	131	13.58	0	0.00		4	3.05	0.07–6.04
Ethnicity (n = 963)	Low land Lao	651	67.60	9	1.38	0.48–2.28	19	2.92	1.62–4.22
	Mid land Lao	248	25.75	6	2.42	0.49–4.34	5	2.02	0.25–3.78
	High land Lao	64	6.65	2	3.13	0.00–7.51	3	4.69	0.00–10.01
1Transportation (n = 939)	on foot	298	31.74	1	0.34	0.00–1.00	6	2.01	0.41–3.62
	bicycle	14	1.49	0	0.00		0	0.00	
	motor bike	364	38.76	7	1.92	0.51–3.34	10	2.75	1.06–4.43
	car	183	19.49	5	2.73	0.35–5.12	6	3.28	0.67–5.88
	hand tractor	66	7.03	3	4.55	0.00–9.71	4	6.06	0.15–11.97
	other	14	1.49	0	0.00		0	0.00	
^2^Time (n = 901)	< 5 minutes	31	3.44	0	0.00		1	3.23	0.00–9.81
	5 to 15 minutes	274	30.41	3	1.09	0.15–2.33	6	2.19	0.45–3.93
	15 to 30 minutes	231	25.64	5	2.16	0.27–4.06	11	4.76	2.00–7.53
	30 to 60 minutes	209	23.20	5	2.39	0.30–4.48	4	1.91	0.04–3.79
	> 60 minutes	156	17.31	3	1.56	0.00–4.68	4	2.56	0.06–5.07
^3^Education (n = 962)	did not finish primary school	307	31.91	7	2.28	0.60–3.96	12	3.91	1.73–6.09
	primary school	374	38.88	5	1.34	0.17–2.51	10	2.67	1.03–4.32
	junior high	185	19.23	3	1.62	0.00–3.46	2	1.08	0.00–2.59
	high school	73	7.59	0	0.00		1	1.37	0.00–4.10
	college/univ	20	2.08	1	5.00	0.00–15.47	2	10.00	0.00–24.41
	other or unknown	3	0.31	1	33.33	0.00–100.00	0	0.00	
^4^Occupation (n = 963)	farmer	683	70.92	13	1.90	0.88–2.93	19	2.78	1.55–4.02
	fisherman	5	0.52	0	0.00		0	0.00	
	laborer	92	9.55	1	1.09	0.00–3.25	5	5.43	0.71–10.16
	public officer	88	9.14	1	1.14	0.00–3.40	3	6.25	1.70–10.80
	factory employee	8	0.83	0	0.00		0	0.00	
	general employee	16	1.66	1	6.25	0.00–19.57	0	0.00	
	merchant	63	6.54	1	1.59	0.00–4.76	0	0.00	
	others	8	0.83	0	0.00		0	0.00	
Mother's surgery (n = 962)	yes	95	9.88	2	2.11	0.00–5.05	3	3.16	0.00–6.74
	no	867	90.12	15	1.73	0.86–2.60	24	2.77	1.67–3.86
Child's sex (n = 960)	male	474	49.38	9	1.89	0.67–3.13			
	female	486	50.63	7	1.44	0.38–2.50			
Place of delivery (n = 961)	province hospital	207	21.54	4	1.93	0.04–3.82	6	2.90	0.59–5.20
	district hospital	105	10.93	2	1.90	0.00–4.56	5	4.76	0.62–8.90
	health center	10	1.04	0	0.00		0	0.00	
	private clinic	11	1.14	0	0.00		1	9.09	0.00–29.35
	at home	569	59.21	8	1.41	0.44–2.38	14	2.46	1.18–3.74
	in the forest	56	5.83	3	5.36	0.00–11.44	1	1.79	0.00–5.36
	other health facility	3	0.32	0	0.00		0	0.00	
Child's surgery (n = 960)	yes	22	2.29	0	0.00				
	no	938	97.71	16	1.71	0.88–2.54			

1 Transportation to the nearest health facility, ^2^ Time to the nearest health facility, ^3^ Mothers' completed education, ^4^ Family head's occupation.

### HBsAg prevalence among the general population

Of the 965 pairs included in the study, 17 children and 27 mothers were positive for HBsAg. Six child and mother pairs were HBsAg positive. The estimated prevalence was 1.7% for children (95% CI: 0.8%–2.6%) and 2.9% for mothers (95% CI: 1.7%–4.2%) after taking the sampling design and weight of each sample into account. HBsAg prevalence did not change significantly between DPT-HepB3 high and low coverage districts in both children and mothers (Table 2).

**Table 2:** HBsAg prevalence among children (5 to 9 years old) and mothers (15 to 45 years old).

	Children's HBsAg (+)	%	95% CI	Standard error	Design effect	Mothers' HBsAg (+)	%	95% CI	Standard error	Design effect
High coverage districts (n = 486)	6	1.14	0.23–2.04	0.44	0.82	18	3.79	1.79–5.79	0.97	1.24
Low coverage districts (n = 479)	11	2.39	0.75–4.03	0.79	1.27	9	1.88	0.49–3.37	0.69	1.22
Total (n = 965)	17	1.72	0.81–2.63	0.44	1.10	27	2.93	1.65–4.20	0.61	1.28

### Potential risk factors

To determine whether background characteristics affect HBsAg status, we conducted multivariate logistic regression analysis in children and their mothers. In children, the mother's HBsAg status was positively associated with hepatitis B infection (Table 3), whereas the other potential risk factors were not associated according to the adjusted odds ratio. We did not obtain information regarding the type of delivery, and we did not find significant differences in HBsAg prevalence associated with delivery settings. No independent factor was positively associated with HBsAg positivity in mothers, according to the adjusted odds ratio (Table 4).

**Table 3:** Unadjusted and adjusted odds ratio for being HBsAg positive among children from five to nine years old in Lao PDR by selected background characteristics.

		Unadjusted odds ratio	95% CI	p	Adjusted odds ratio	95% CI	p
DPT3 coverage	high	1(reference)					
	low	2.13	0.73–6.21	0.16	3.47	0.77–15.64	0.10
Mothers' age	15 to 29	1(reference)					
	30 to 45	0.70	0.28–1.78	0.44	0.87	0.31–2.47	0.79
Ethnicity	Low land Lao	1(reference)					
	others	1.90	0.67–5.40	0.22	1.41	0.26–7.72	0.68
Education	none	1(reference)					
	finished primary school or upper	1.50	0.67–3.36	0.30	1.03	0.27–3.89	0.96
Occupation	white collar	1(reference)					
	blue collar	1.15	0.37–3.64	0.80	0.60	0.18–1.96	0.38
Sex	male	1(reference)					
	female	0.75	0.21–2.62	0.63	0.65	0.21–2.08	0.46
Birth place	health facility	1(reference)					
	non-health facility	0.98	0.39–2.49	0.97	0.79	0.28–2.21	0.64
Mothers' HBsAg	negative	1(reference)					
	positive	24.02	9.45–61.07	0.00	28.13	10.21–77.53	0.00

**Table 4:** Unadjusted and adjusted odds ratio for being HBsAg positive among mothers from 15 to 45 years old in Lao PDR by selected background characteristics.

		Unadjusted odds ratio	95% CI	p	Adjusted odds ratio	95% CI	p
DPT3 coverage	high	1(reference)					
	low	0.50	0.20–1.28	0.14	0.47	0.19–1.16	0.10
Mothers' age	15 to 29	1(reference)					
	30 to 45	1.03	0.43–2.51	0.94	0.94	0.39–2.25	0.88
Ethnicity	Low land Lao	1(reference)					
	others	0.80	0.30–2.17	0.65	0.68	0.25–1.85	0.44
Education	none	1(reference)					
	finished primary school or upper	1.68	0.70–4.01	0.23	2.04	0.89–4.68	0.09
Occupation	white collar	1(reference)					
	blue collar	1.71	0.53–5.55	0.35	1.93	0.68–5.50	0.21
History of surgery	no	1(reference)					
	yes	1.28	0.39–4.25	0.67	1.30	0.35–4.78	0.68

### Immunization status

Written immunization records were available for 213 out of 965 children (22.1%). One hundred ninety eight children were vaccinated with three doses of hepatitis B vaccine, and 34 children were immunized on the day of birth or the following day. Five out of 213 children with immunization records were HBsAg positive (2.35%; 95% CI: 0.30–4.40%), while 12 of 752 without immunization records were HBsAg positive (1.60%; 95% CI: 0.70–2.49%). The differences between the two groups were not significant (*p* = 0.46).

## Discussion

### HBsAg prevalence among the general population

The estimated HBsAg prevalence in the general population was much lower in both children and adults than that of previous reports from neighboring countries and Lao PDR. For example, HBsAg prevalence in adults in Cambodia, Thailand, and Vietnam was reported to be 7.7% (95% CI: 6.2%–9.3%) [12], 6 to 10% [15], [20], and 18.8% (95% CI: 15.7%–21.9%) [13], respectively. Data on HBsAg prevalence amongst children was relatively scarce, and reported to be 3.5% (95% CI: 2.4%–4.8%) in Cambodia [21], and 18.4% (95% CI: 13.4%–23.4%) in Vietnam [13]. In Lao PDR, studies in blood donors, hospitalized patients, and Lao migrant workers tested in Thailand showed HBsAg prevalence of 8.73% (95% CI: 8.69%–8.77%) [22], 17.99% (95% CI: 17.81%–18.17%) [23], and 6.86% (95% CI: 6.80%–6.92%) [24] based on the given numerators and denominators in the articles, respectively.

Since the study objective was to estimate the nationwide HBsAg prevalence among the general population of Lao PDR, and thus the study design is a cross sectional survey, it is difficult to explain the reasons for the unexpectedly low prevalence. There are several potential explanations for this observation. The survey methodology used was very different from that used for blood donors, patients, and migrant workers. We used probability sampling and thus the results are representative of the whole population, whereas studies of blood donors, hospitalized patients, and migrant workers used non-probability sampling and therefore the results are restricted to these populations. The primary objective of our survey was to estimate HBsAg prevalence among the general population, so probability sampling was the most appropriate choice. Demographic conditions among the sampled population are determined by survey methodology, and therefore the results showed discrepancy. The WHO strongly recommends probability sampling for hepatitis B prevalence survey [7], [15], [17]. Although Lao PDR has the lowest population density of the Indochina peninsula countries [25], the precise effects on hepatitis B prevalence of the reduced frequency of human to human contact due to the country's relatively low population density and less developed infrastructure remain unclear.

The number of HBsAg positives varied from 0 to 4 per cluster. Since the sampling design of the survey aimed to estimate the prevalence in the whole country, it is difficult to determine whether these differences reflect the local endemic status.

### Potential risk factors

Our survey revealed that no potential risk factors were significantly associated with the children's infection status, with the exception of the mothers' hepatitis B infection status. HBsAg prevalence surveys in other countries revealed that history of surgery [26], [27], level of education [26], and ethnicity [28] were independently associated with hepatitis B infection. The reason why we could not find any potential risk factors positively associated with hepatitis B infection among children is not clear. However, it should be noted that the primary objective of the present study was to assess HBsAg prevalence, and not its risk factors. Additionally, some reports found that HIV positive individuals are positively associated with hepatitis B virus infection [29], [30]; however, we did not investigate HIV due to limited budget.

### WHO's regional target

The interim target of the WHO is to reduce HBsAg prevalence to less than 2% in children aged at least 5 years old by 2012 [7], [31]. The point prevalence is used for monitoring the control of hepatitis B. The Regional Office for the Western pacific recommended that the country conduct a national HBsAg prevalence survey to verify whether the country has reached the regional prevalence target [9]. Following these criteria, Lao PDR had already achieved its goal. However, it is unlikely that Lao PDR achieved the target through the immunization program alone because the country has the lowest immunization coverage of all countries in the region [7], [9]. Considering the relatively lower HBsAg seroprevalence among the mothers compared to those reported in previous studies, it is likely that Lao PDR had a lower prevalence even before the introduction of the hepatitis B immunization program. Therefore, the final target of reducing HBsAg prevalence to less than 1% in children aged at least 5 years could be difficult to achieve if the country simply continues its current immunization policy.

A nationwide prevalence survey targeting the general population is ideally conducted before implementing the immunization strategy to evaluate hepatitis B epidemiology. However, we were able to understand the epidemiology to some degree, even after implementation of immunization policy, because adults usually represent the pre-vaccination era [15], [17].

### Strengths of the study

The present study is the first nationwide survey on the prevalence of hepatitis B in the general population both before and after the implementation of a hepatitis B immunization policy in Lao PDR and other Southeast Asian countries. We applied multistage stratified cluster sampling to better represent the general population. The design effect of prevalence was calculated between 0.8 and 1.3, which was acceptable as we set it around 2.0 before the survey.

The background characteristics of our sampled population were similar to those of another nationwide population-based study, the Lao PDR Reproductive Health Survey (LRHS) [32] conducted in 2005. For example, the locations of current residence (north, central, and south) were 33.3%, 41.7%, and 25.0% in our survey, and 38.6%, 38.9%, and 22.5% in the LRHS. The levels of mothers' completed education (none, primary school, secondary school or more) were 31.9%, 38.9%, and 29.2% in our survey, and 28.8%, 43.7%, and 27.5% in the LRHS. The LRHS applied the multistage stratified cluster sampling method and surveyed more than 13,000 women all over the country. A direct comparison of the populations sampled by the two different surveys is difficult to perform as the primary objectives were different. Despite this, our sampled population is considered to likely represent the general population in Lao PDR.

### Limitations of the study

There are several limitations in our study that should be addressed. First, the population data is based on the census conducted in 2005. After 2005, the population distribution may have changed and some of the villages could have merged, thereby creating bias in the findings. Fortunately, we did not survey any villages that disappeared or merged.

Second, floating or marginal populations are likely to be missed from the residential lists, and these populations could be a source of HIV and hepatitis B virus infections [33]. In future seroprevalence surveys, these subpopulations should be accounted for by using specific approaches, such as oversampling.

Third, population immunity levels were difficult to measure or estimate. The possession of immunization certificates was low, because many participants had already finished their scheduled vaccinations before 12 months of age, and relevant documents were lost. In the present study, we did not have enough data from health centers due to time and budget limitations. Since we did not examine immunization markers, such as HBsAb, herd immunity levels are unknown.

Lastly, adult men were not included in the survey. Serological studies in the past indicated that men have higher HBsAg rates than women [8], [21], [28]. In Lao PDR, male blood donors presented with 9.7% HBsAg positive prevalence, while the prevalence in females was 6.2% [22]. When considering the disease burden of hepatitis B virus infections, it is better to include both sexes [26].

To the best of our knowledge, this is the first nationwide, population-based serological survey on chronic hepatitis B virus infections both before and after implementation of hepatitis B immunization in Southeast Asia, where disease burden is high. As such, our results provide valuable information on a hepatitis B immunization program and a useful baseline against which to compare future assessments in this region.

National immunization policy should be based on the disease epidemiology [3]. However, in Southeast Asia, understanding of the epidemiology of hepatitis B remains unsatisfactory. Even when a country implements a hepatitis B immunization program for children and the prevalence of disease reaches the target (i.e., less than 2% among children aged 5 years or older), we cannot conclude that the immunization program alone contributed to reduced disease prevalence without comparing it to the disease prevalence in the pre-vaccine generation, i.e., adults. Nationwide surveys assessing disease prevalence in the generations before and after the implementation of a vaccination program will provide valuable information for understanding hepatitis B epidemiology. Therefore, we recommend surveying hepatitis B seroprevalence in both generations.

## Conclusions

We determined the nationwide HBsAg prevalence among children (1.7%; 95% CI: 0.8%–2.6%) and their mothers (2.9%; 95% CI: 1.6%–4.2%) in Lao PDR. This is the first report to estimate the nationwide prevalence of chronic hepatitis B in pre- and post-hepatitis B immunization generations in Southeast Asia, where hepatitis B infections are a substantial burden. The estimated prevalence was below that of previous studies, suggesting that our understanding of this disease's epidemiology is lacking and warrants further investigation. We recommend that the prevalence among the pre- and post-vaccine eras should be investigated when conducting hepatitis B seroprevalence surveys.
